# Lectin binding to cutaneous malignant melanoma: HPA is associated with metastasis formation

**DOI:** 10.1054/bjoc.2000.1673

**Published:** 2001-03

**Authors:** A Thies, I Moll, J Berger, U Schumacher

**Affiliations:** Institute for Anatomy, University Hospital Hamburg-Eppendorf, Martinistrasse 52, Hamburg, D-20246; Dermatological Hospital, University-Hospital Hamburg-Eppendorf, Martinistrasse 52, Hamburg, D-20246; Institute of Mathematics and Computer Science in Medicine, University Hospital Hamburg-Eppendorf, Martinistrasse 52, Hamburg, D-20246

**Keywords:** melanoma, lectins, metastasis, prognosis, HPA

## Abstract

Changes in protein glycosylation of tumour cells, as detected by lectin histochemistry, have been associated with metastasis formation in several human malignancies. This study analysed the association between lectin binding and metastasis in cutaneous malignant melanoma. In a 10-year retrospective study, sections of 100 primary cutaneous malignant melanomas were histochemically stained for the following 5 lectins: HPA, SNA-I, MAA, WGA and PHA-L, differing in their carbohydrate specificity. Since differences in the results of HPA binding depending on methodology have been reported, an indirect and a biotinylated method were employed for HPA. Kaplan–Meier analysis of time to first metastasis revealed a positive correlation between HPA binding and metastasis for both methods, with the biotinylated HPA method (*P*< 0.0001) being superior to the ‘indirect’ method (*P* = 0.0006). Cox regression analysis demonstrated that even after adjustment for stage, HPA positivity is an independent predictor for metastasis. The results of the present study indicate that *N* -acetyl-galactosamine/-glucosamine residues, recognized by HPA, are linked to metastasis in malignant melanoma. In contrast, β1-6 branched oligosaccharides or sialic acid residues, both of which were correlated with metastasis in other malignancies, are of no functional importance for metastasis formation in malignant melanoma. Thus, HPA proved to be a useful and independent prognostic marker for the metastatic phenotype of melanoma. © 2001 Cancer Research Campaign http://www.bjcancer.com


*British Journal of Cancer* (2001) **84**(6), 819–823
© 2001 Cancer Research Campaign
doi: 10.1054/ bjoc.2000.1673, available online at http://www.idealibrary.com on IDEAL®

http://www.bjcancer.com

### Lectin binding to cutaneous malignant melanoma: HPA is associated with metastasis formation

**A Thies[1], I Moll[2], J Berger[3] and U Schumacher[1]**

[1]Institute for Anatomy, University Hospital Hamburg-Eppendorf, Martinistrasse 52, D-20246 Hamburg; [2]Dermatological Hospital, University-Hospital Hamburg-Eppendorf, Martinistrasse 52, D-20246 Hamburg; [3]Institute of Mathematics and Computer Science in Medicine, University Hospital Hamburg-Eppendorf, Martinistrasse 52, D-20246 Hamburg

**Summary** Changes in protein glycosylation of tumour cells, as detected by lectin histochemistry, have been associated with metastasis formation in several human malignancies. This study analysed the association between lectin binding and metastasis in cutaneous malignant melanoma. In a 10-year retrospective study, sections of 100 primary cutaneous malignant melanomas were histochemically stained for the following 5 lectins: HPA, SNA-I, MAA, WGA and PHA-L, differing in their carbohydrate specificity. Since differences in the results of HPA binding depending on methodology have been reported, an indirect and a biotinylated method were employed for HPA. Kaplan–Meier analysis of time to first metastasis revealed a positive correlation between HPA binding and metastasis for both methods, with the biotinylated HPA method ($P < 0.0001$) being superior to the 'indirect' method ($P = 0.0006$). Cox regression analysis demonstrated that even after adjustment for stage, HPA positivity is an independent predictor for metastasis. The results of the present study indicate that *N*-acetyl-galactosamine/-glucosamine residues, recognized by HPA, are linked to metastasis in malignant melanoma. In contrast, β1-6 branched oligosaccharides or sialic acid residues, both of which were correlated with metastasis in other malignancies, are of no functional importance for metastasis formation in malignant melanoma. Thus, HPA proved to be a useful and independent prognostic marker for the metastatic phenotype of melanoma. © 2001 Cancer Research Campaign http://www.bjcancer.com

**Keywords**: melanoma; lectins; metastasis; prognosis; HPA

Despite progress in molecular biology, the prognosis of patients suffering from cutaneous malignant melanoma, remains unsatisfactory (Marks and Kopf, 1995). This poor outlook is due to the fact that once the tumour has reached the metastatic state, no curative treatment is available. To achieve real progress in the treatment of melanoma, the process of metastatic formation has to be understood, in order to provide a rational approach for its therapy.

Metastasis is a multistep phenomenon, which involves the loosening of the tumour cells from the primary tumour, the degradation of the extracellular matrix and the invasion of the blood vessels at the site of the primary tumour. Having reached the bloodstream, the putative metastatic cells have to survive within the circulation and have to attach to the endothelium at the future metastatic site. There they have to penetrate the endothelium, degrade the basal lamina and grow at the distant site (Hart et al, 1989; Hart and Saini, 1992; Engers and Gabbert, 1998). Several of these steps involve cell-to-cell and/or cell-to-matrix interactions. Therefore it is of particular interest to study the external surface of the malignant cells, which mediates these interactions.

The outer surface of all mammalian cells, including tumour cells, is covered by a carbohydrate rich coat, the glycocalix. The composition of this carbohydrate coat has a profound influence on the cells' behaviour and changes in the sugar composition have been associated with metastasis formation (Mitchell and Schumacher, 1999). Changes in carbohydrate composition can be analysed using lectins, carbohydrate-binding proteins of non-immunological origin. One lectin in particular, *Helix pomatia* agglutinin (HPA), has been extensively used in cancer research as an indicator for poor prognosis. An association between HPA binding to the cells of the primary tumour and a poor prognosis has been shown for breast, colorectal, gastric and prostate cancer (Leathem et al, 1985; Leathem and Brooks, 1987; Kakeji et al, 1991; Schumacher et al, 1994; Shirashi et al, 1992). Because of the biological significance of these carbohydrate residues, the binding of several lectins, each having a different carbohydrate specificity, will be tested for its prognostic value in malignant melanoma in this study. In this lectin-binding analysis, particular emphasis will be placed upon the usage of 2 different methods for HPA staining as the methodology strongly influences the prognostic value of the staining (Brooks et al, 1996). 2 different methods for HPA binding, a biotinylated HPA method, and an indirect method using native HPA and an anti-HPA antibody, will be compared.

### MATERIALS AND METHODS

Paraffin wax sections of human cutaneous malignant melanoma from 123 patients, who underwent surgery between 1983 and 1996 in the Dermatological Hospital, University Hospital Eppendorf, Hamburg, Germany, were investigated. The data from 100 patients could be traced in the outpatients clinic and was followed up for 10 years. Dates of diagnosis, surgery, occurrence of first metastasis and death and data of Clark's level of invasion, Breslow's vertical tumour thickness and clinical stage were recorded from the original reports. Informed consent of all patients, whose tumours were investigated, had been obtained following institutional guidelines.

*Received 7 August 2000*
*Revised 20 November 2000*
*Accepted 30 November 2000*

*Correspondence to*: A Thies

The majority of the patients were female (female = 60; male = 40) and the median age was 66.5 years (range 19–97 years). 41 patients presented with stage IA tumours, 22 with IB, 32 with IIA and 5 with IIB tumours; staging was performed according to the recommendations of the German Dermatological Society (DDG), (Orfanos et al, 1994). After a median follow up of 10 years, 34 patients showed clinical signs of metastasis and of these 20 had died of metastatic disease.

### Lectin histochemistry

Deparaffinized, 5 µm thick sections were rehydrated through a series of graded ethanols and incubated with 0.1% trypsin (Biochrom KG, Berlin, Germany) in Tris-buffered saline with calcium chloride (1 mM) and magnesium chloride (1 mM) (Merck, Darmstadt, Germany) added (‘lectin buffer’, pH 7.6) for 15 min at 37˚C. After careful washes under running tap water, sections were transferred into lectin buffer, washed 3 times and then incubated with 10 µg $ml^{-1}$ biotinylated lectin (HPA, PHA-L, MAA, SNA-I, and WGA; Sigma, Darmstadt, Germany), respectively, for 1 h at room temperature. (For lectins, their abbreviation and carbohydrate specificity, see Table 1.) After careful washes in Tris buffered saline (TBS pH 7.6), an incubation with a streptavidin-alkaline phosphatase complex (Vectastain, Vector, ABC kit, Burlingame, CA) for 30 min and further washes in TBS followed. Alkaline phosphatase enzyme reactivity was visualized using Naphtol-AS-biphosphate as substrate and hexatozised New Fuchsin was used for simultaneous coupling. Slides were counterstained with Mayer’s hemalum solution 1:1 in distilled water for 10 s, blued under running tap water and finally mounted with Crystal Mount (Biomeda, Foster City, CA).

For HPA, an additional, indirect staining method, following a standard protocol described earlier (Brooks et al, 1996) was performed. The only variation to the described method by Brooks et al (1996) was, that the binding sites were visualized using an alkaline phosphatase complex instead of a peroxidase complex. Slides were pre-treated as described above, but were then incubated with native unconjugated HPA (10 µg $ml^{-1}$) for 1 h at room temperature and further washes in lectin buffer followed. After an incubation with 10% normal swine serum (DAKO, Glostrup, Denmark) for 30 min at room temperature, slides were incubated overnight with a 1:100 diluted rabbit anti-HPA antibody (EY Lab, San Mato, CA) at 4˚C. The next morning, the slides were incubated with a 1:300 diluted biotinylated swine anti-rabbit antibody (DAKO, Glostrup, Denmark) for 30 min, followed by careful washes in lectin buffer. This was followed by an incubation with a streptavidin-alkaline phosphate complex (Vectastain, Vector, ABC kit, Burlingame, CA) for 30 min and additional washes in TBS. Developing of the enzyme reactivity, counterstaining and mounting was performed as described above. For both staining methods, control sections were treated the same way omitting the lectin incubation. As a positive control and for assuming staining consistency, one case that previously stained strongly for lectin binding was included with every batch of staining.

**Table 1**

| Lectin | Abbreviation | Carbohydrate specificity |
|---|---|---|
| *Helix pomatia* | HPA | D-galNAc > glcNAc |
| *Maackia amurensis* | MAA | α-2,3 branched sialic acid |
| *Sambucus nigra* | SNA-I | α-2,6 branched sialic acid |
| *Phaseolus vulgaris* | PHA-L | complex carbohydrates |
| *Triticum vulgaris* | WGA | β-D-glcNAc, β-D-glcNAc NeuAc, galNAc > lac > gal > galNAc |

Range of used lectins, their abbreviation and carbohydrate specificity. Carbohydrate specificity is listed in decreasing affinity. galNAc = *N*-Acetylgalactosamine; glcNAc = *N*-Acetylglucosamine; NeuAc = neuraminic acid; gal = galactose; glc = glucose.

### Histological evaluation and statistical analysis

For histological evaluation of the staining result, the definition of Leathem et al (1985) was adhered to: ‘negative’ (–) indicated no staining or very weak staining of single tumour cells less than 5%, regardless of staining pattern or staining intensity. Regular supranuclear staining (Golgi field) of whole clusters of the melanoma was also regarded as negative. This was done based upon works on HPA-binding pattern in human colorectal cancer (Schumacher et al, 1994), where this pattern of reactivity proved to be of no prognostic value. ‘Positive’ indicated that at least 6% of the tumour cells were stained. Microscopic evaluation was carried out by 2 independent, blinded observers. The observers agreed in 95% of the cases; in the remaining cases consensus was achieved after discussion. The intra-observer reproducibility exceeded 97%. The slides were examined under a Zeiss Axioplan photomicroscope and photographed with a Kodak Ektachrome 64T colour film. Statistical analysis was carried out, preparing survival curves according to the Kaplan–Meier method (Kaplan and Meier, 1958) for time to first metastasis, unadjusted for clinical stage, for each of the 5 lectins, using Graph Pad Prism (Intuitive Software for Science, San Diego, CA) on an IBM-compatible microcomputer. Furthermore, Cox-regression analysis for HPA binding adjusted for clinical stage were performed using SPSS for Windows version 9 (SPSS Inc, Chicago, Illinois, USA).

### RESULTS

### Lectin histochemistry

A summary of all staining results is given in Table 2.

HPA staining exhibited a very homogenous staining pattern with both methods: either the far majority of tumour cells were stained or no staining of the melanoma cells could be detected (see Figure 1). Staining intensity of the indirect method was more intense than of the biotinylated HPA method. The biotinylated HPA method detected 25 HPA-positive tumours, of which 17 had metastasized, while the indirect method revealed 40 HPA-positive tumours, of which 21 had metastasized. Comparing the staining results of the both methods for HPA, 17 tumours were positive in both methods and of these 13 had metastasized.

PHA-L exhibited heterogeneous intensity of tumour cell staining within individual melanomas. 70 melanomas, of which 22 had metastasized, contained PHA-L-positive tumour cells. Evaluation of binding pattern of SNA-I revealed 42 positive melanomas. 17 of the 42 SNA-I-positive melanomas and 17 of the 58 SNA-I-negative tumours had metastasized. As to the staining characteristics of SNA-I, it was striking to note that only single clusters of tumour cells were positive within one melanoma. MAA did not bind to melanoma cells at all, in all 100 cases and WGA exhibited constant positive staining of all melansomas.

 © 2001 Cancer Research Campaign

**Table 2**

| Lectin | *P* value | Staining pattern of tumour cells | Metastasized / not metastasized |
|---|---|---|---|
| HPA | | 25 positive | 17 / 8 |
| biotin | < 0.0001 | 75 negative | 17 / 58 |
| HPA | | 40 positive | 21 / 19 |
| indirect | 0.0006 | | |
| | | 60 negative | 13 / 47 |
| MAA | ns | no binding at all | |
| PHA-L | ns | 70 positive | 22 / 48 |
| | 0.44 | | |
| | | 30 negative | 12 / 18 |
| SNA-I | ns | 42 positive | 17 / 25 |
| | 0.15 | | |
| | | 58 negative | 17 / 41 |
| WGA | ns | all positive | |

ns = not significant

### Survival analysis

### *Univariate*

Analysis of HPA binding versus no binding was significantly correlated with metastasis: those patients whose primary melanomas bound HPA, had a significantly higher risk of subsequent metastasis than those patients whose tumours did not bind this lectin (see Figure 2). Both methods gave excellent correlation with subsequent metastasis. Although, in distinguishing between the metastatic and the non-metastatic subgroup, the biotinylated HPA method ($P < 0.0001$) was slightly superior to the indirect method ($P = 0.0006$). Kaplan–Meier analysis detected no correlation between MAA, SNA-I, PHA-L and WGA binding to melanoma cells and metastasis formation.

### *Multivariate*

A Cox-regression model including HPA-binding status and tumour stage coded as indicator variable demonstrated that HPA positivity is an independent predictor for metastasis. For patients whose tumours are HPA positive, the risk of metastasis is 2.6 times higher than for patients with HPA negative tumours (95%CI:1.3–5.5) (see Table 3).

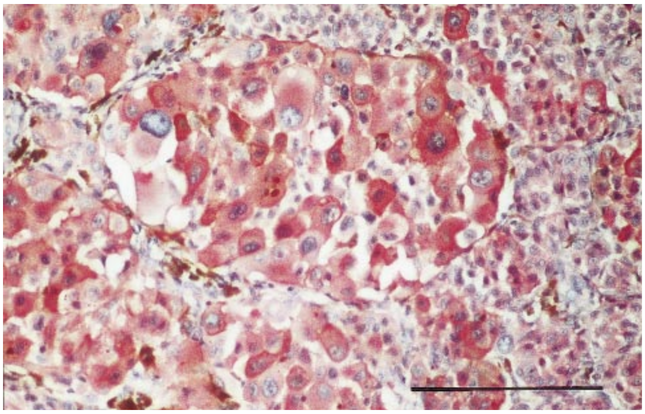

**Figure 1** Intense (++) to very intense (+++) HPA staining of malignant melanoma cells (indirect method). Note that the far majority of tumour cells is stained. Very intense staining of the cell membrane and additional cytoplasmic staining of the metastatic phenotype of malignant melanoma. Magnification x 375. Line represents 100 µm

**Table 3** Simultaneous influence of HPA and stage on the metastatis rate. Result of the Cox regression; hazard ratio and 95% confidence interval

| Variable | Exp(B) | Lower | Upper |
|---|---|---|---|
| HPA | 2.62 | 1.25 | 5.48 |
| STAGE (1) | 3.43 | 1.12 | 10.51 IB versus IA |
| STAGE (2) | 4.78 | 1.73 | 13.15 IIA versus IA |
| STAGE (3) | 11.33 | 2.92 | 43.88 IIB versus IA |

### DISCUSSION

The aim of the present study was twofold: first, a possible correlation between the expression of lectin-binding sites in cutaneous malignant melanoma and the development of metastasis was investigated and second, as the methodology strongly influences the prognostic value of the staining (Brooks et al, 1996), 2 different methods for HPA binding, one method, using biotinylated HPA, and an indirect method, using native HPA and an anti-HPA antibody, were compared.

Of the 5 lectins tested, binding of HPA was strongly correlated with metastasis in this patient series. Multivariate analysis demonstrated that HPA binding to primary malignant melanomas is an independent marker for the risk of metastasis; patients with a HPA-positive melanoma have a 2.6 times higher risk for metastasis over patients with HPA-negative melanomas. Hence, HPA positivity is an additional tumour cell characteristic beneath the classical staging using tumour thickness, which places patients into 4 risk groups. Therefore assessment of HPA-binding characteristics would be one approach to more precisely identify the metastatic phenotype of malignant melanoma. However, the prognostic relevance of HPA now has to be validated in a bigger patient cohort, especially when discussing prognostic markers in malignant melanoma (Doré et al, 1997), but the results in our series of 100 patients clearly prove that HPA positivity is strongly correlated with metastatic spread.

HPA binding to the metastatic subtype of melanoma is not only a useful histochemical marker for a high risk of subsequent metastasis but also might be functionally associated with the metastatic processes. The results of the present study strongly support the previous hypothesis that expression of *N*-acetyl-galactosamine (GalNAc)/-glucosamine (GlcNAc) residues on the cell surface of malignant cells, as detected by HPA binding, is involved in the complex process of metastatic spread. The biosynthetic pathway of the HPA binding saccharides is hence of particular interest. Two different ways of biosynthesis are possible: first, a neo-expression of *N*-acetyl-galactosamine/-glucosamine residues on the metastatic melanoma cells or secondly, a loss of terminal carbohydrate residues, unmasking sub-terminal HPA-binding partners could account for their HPA positivity. The latter has been propagated for HPA-binding sites in breast and colon cancers: in breast cancer, the pre-treatment of tissue sections with sialidase destroyed the significance of the HPA binding results (Fenlon et al, 1987), indicating that terminal sialic acid masks HPA-binding sites and is thus involved in the metastatic process. Furthermore, serial analysis of gene expression (SAGE) in colon cancer and normal colonic mucosa resulted in a significant depression of the $\alpha$ 2–3 sialyltransferase transcript in colon cancers (Zhang et al, 1997). The end product of this enzyme activity can be visualized using the $\alpha$ 2–3 sialic acid-specific MAA. However, the simple loss of terminal sialic acids, as discussed above, is not sufficient to explain the association between HPA binding and metastasis in

© 2001 Cancer Research Campaign

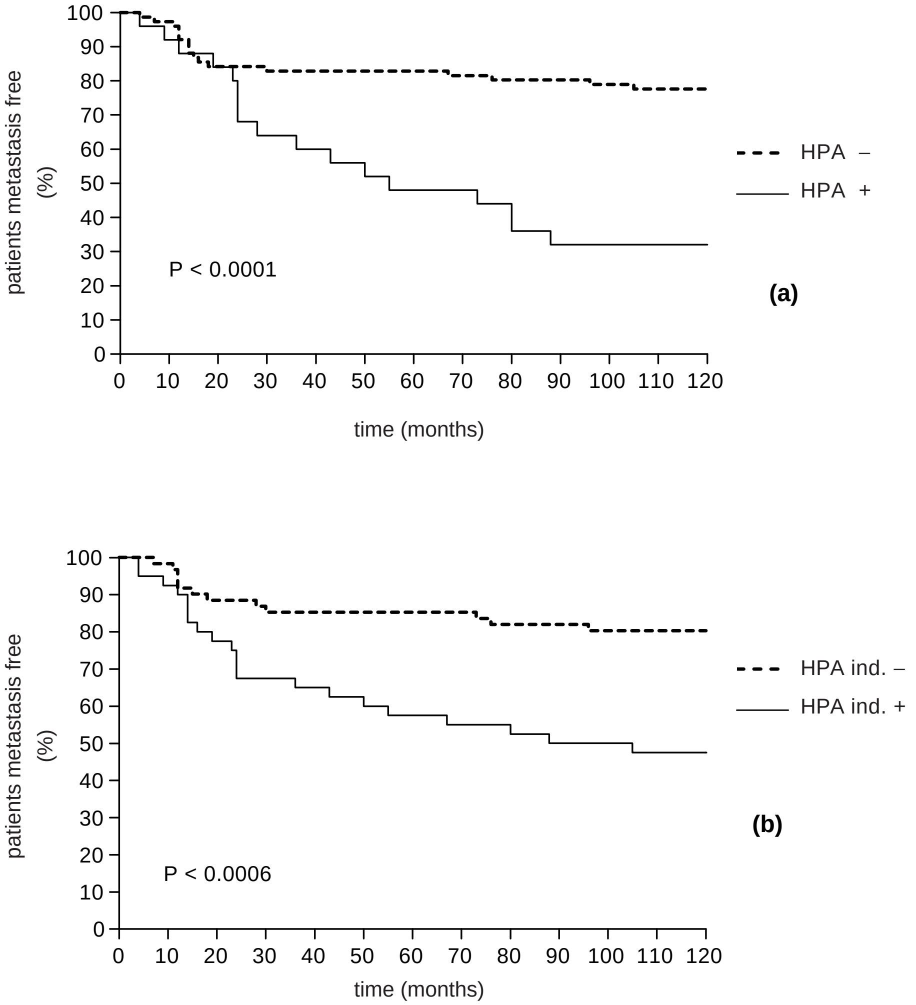

**Figure 2** Kaplan–Meier analysis of time to first metastasis for HPA-stainers (HPA+) versus non-stainers (HPA–); (**a**) biotinylated method and (**b**) indirect method. Those patients, whose tumours expressed HPA-binding sites had a significant higher risk of subsequent metastasis than those patients whose melanomas were HPA negative malignant melanoma, as binding of MAA was not correlated with metastasis. Hence, subtle differences in the carbohydrate residues of breast cancer cells and melanoma cells, originating from different embryonic tissues, are present in the transformation to the metastatic phenotype. Differences in the prognostic value of carbohydrate residues between breast cancer and malignant melanoma are also highlighted by PHA-L. This lectin proved to be of prognostic significance in breast and colon cancers (Fernandes et al, 1991), however, no such significance could be detected in our series of malignant melanomas.

HPA positivity of the metastatic melanomas could not be ascribed to a specific region of the tumour, which is in contrast to a study on the expression of PNA binding sites, where PNA positivity was concentrated at the invading front of the primary melanomas, suggesting a functional role of galactose residues in cell invasion (Cochran et al, 1999). In HPA-positive melanomas, the great majority of tumour cells bound the lectin, so that one could speculate that GalNAc/GlucNAc residues are not involved in tumour invasion, but into later steps of tumour progression such as adhesion to endothelia at the evasion site from the bloodstream.

Although several studies have demonstrated a strong relationship between HPA binding to primary tumours and a poor prognosis in breast (Leathem and Brooks, 1987), oesophageal (Yoshida et al, 1993) gastric (Kakeji et al, 1991), prostate (Shirashi et al, 1992) and colorectal cancers (Ikeda et al, 1994; Schumacher et al, 1994), some controversy over the prognostic significance of HPA binding in different malignancies existed (Walker, 1993) and 2 reports have failed to confirm the prognostic significance of HPA binding to the metastatic phenotype in breast cancer (Galea et al, 1991; Gusterson et al, 1993). As Galea et al (1991) and Gusterson et al (1993) used HPA, which was directly conjugated with horseradish peroxidase and all other reports used native HPA, which was detected using anti-HPA antibodies and immunoperoxidase techniques, it is likely that the different methodology used, resulted in the difference of the results. Indeed, Brooks et al (1996) could show in one study involving 373 patients that the methodology critically influences the outcome of the studies. To further resolve the problem, which methodology is the appropriate one to determine lectin binding, this study compared a biotinylated HPA method and an indirect method for HPA binding in serial sections. To be able to directly compare the effect of biotinylation on lectin binding, a streptavidin-alkaline phosphatase detection kit (ABC kit) was used for both methods, as influences on the sensitivity as a consequence of amplification through a biotin–streptavidin complex have been previously

 © 2001 Cancer Research Campaign described (Hsu et al, 1981). Furthermore, all tissue samples were collected from the same department, where they were routinely fixed and processed in the same way so that influences through different processing, which also influences HPA binding sites (Schumacher et al, 1995), can probably be neglected. Both the classical indirect method ($P$ = 0.0006) and the biotinylated method ($P$ < 0.0001) proved to show excellent correlation between HPA binding and metastasis in malignant melanoma. Consistent with this correlation, the staining of many melanomas was identical with both methods. The biotinylated HPA method with a specificity of 78% was slightly superior to the indirect method with a specificity of 71%. This contrasts with findings reported in breast cancer, where the indirect method showed a strong correlation between binding and long-term survival, whereas the direct method was only weakly correlated with prognosis (Brooks et al, 1996). The authors explained this by the fact that the lectin, used for the indirect method, was conjugated to the comparatively large horseradish peroxidase molecule, which might interfere sterically with the lectin binding. In our study, however, the lectin used for the direct method was conjugated to biotin, which has now been proved to have less influence on the lectin-binding site. Another factor, which might account for the differences between the 2 studies is that alkaline phosphatase was used as a detection molecule, which was necessary as the melanin pigment interferes with the brown reaction product of the horseradish peroxidase. Hence, the suggestion by Brooks et al (1996) that the covalent coupling of HRP to HPA causes alterations of the HPA sugar-binding site is still valid. Altogether the results of this study demonstrate that alteration of the binding characteristics of HPA by biotinylation are not significant and therefore the biotinylated method should be the method of choice for future lectin-histochemical studies.

Comparing the results of this clinical study with result on lectin-binding pattern in the melanoma/nude mouse model, as proposed by Kjionniksen et al (1994), demonstrating that HPA-positive human melanoma cell lines metastasize spontaneously in nude mice, while HPA-negative cell lines do not, appropriately reflects the clinical situation. In contrast, the mouse melanoma study by Tao and Burger (1982), implicating that WGA-binding sites expressed on melanoma cells play a role in metastasis formation, are incongruent with the findings in our clinical study, where WGA was not of prognostic significance. Hence, the mouse melanoma models are not directly transferable to a clinical situation with respect to the carbohydrate expression pattern.

In conclusion, the results of the present study prove that HPA is a reliable and independent prognostic marker for the metastatic potential in human cutaneous malignant melanoma. In contrast, complex type oligosaccharides and sialic acid residues, which have been implicated to play a functional role in metastasis formation in other malignancies, are of no importance for the metastatic spread in this tumour entity.

### ACKNOWLEDGEMENT

The financial support from the Gustav Spierling Stiftung (Hamburg, Germany) is gratefully acknowledged.

### REFERENCES

Brooks SA, Lymboura M, Schumacher U and Leathem AJ (1996) Histochemistry to detect *Helix pomatia* lectin binding in breast cancer: methodology makes a difference. *J Histochem Cytochem* **44**: 519–524

Cochran AJ, Wen DR, Berthier-Vergnes O, Bailly C, Dore JF, Berard F, Moulin G and Thomas L (1999) Cytoplasmic accumulation of peanut agglutinin-binding glycoconjugates in the cells of primary melanoma correlates with clinical outcome. *Hum Pathol* **30**: 556–561

Doré JF, Maisonneuve P, Cattaruzza MS, Autier P, Cochran AJ and Boyle P (1997) A molecular epidemiological approach to the study of expression of a metastasis marker in primary melanomas and ist correlation with individual patient's risk of recurrence or metastasis. *Melanoma Res* **7** (suppl.2): 121–125

Engers R and Gabbert HE (1998) Basic mechanisms of metastasizing. Fundamental of tumor biology and clinical implications. *Onkologe* **4**: 682–688

Fenlon S, Ellis IO, Bell J, Todd JH, Elston CW and Blamey RW (1987) Helix pomatia and Ulex europaeus lectin binding in human breast carcinoma. *J Pathol* **152**: 169–176

Fernandes B, Sagman U, Auger M, Demitrio M and Dennis JW (1991) β1-6 branched oligosaccharides as a marker of tumor progression in human breast and colon neoplasia. *Cancer Res* **51**: 718–723

Galea MH, Ellis IO, Bell J, Elston CW and Blamey RW (1991) Prediction of lymph node involvement in breast cancer. *Lancet* **338**: 392–393

Gusterson BA, and the International (Ludwig) Breast Cancer Study Group (1993) Prognostic value of *Helix pomatia* in breast cancer. *Br J Cancer* **68**: 146–150

Hart IR and Saini A (1992) Biology of tumour metastasis. *Lancet* **339**: 1453–1461

Hart IR, Goode T and Wilson GR (1989) Molecular aspects of the metastatic cascade. *Biochem Biophys Acta* **989**: 65–84

Hsu SM, Raine L and Fanger H (1981) Use of Avidin-Biotin-Peroxidase Complex (ABC) in Immunoperoxidase Techniques: A comparison between ABC and Unlabeled Antibody (PAP) Procedures. *J Histochem Cytochem* **29**(4): 577–580

Ikeda Y, Mori M, Adachi Y, Matsushima T and Sugimachi K (1994) Prognostic value of the histochemical expression of *Helix pomatia* agglutinin in advanced colorectal cancer. *Dis Colon Rectum* **37**: 181–184

Kakeji Y, Tsujitani S, Mori M, Maehara Y and Sugimachi K (1991) *Helix pomatia* binding activity is a predictor of survival time for patients with gastric carcinoma. *Cancer* **68**: 2438–2442

Kaplan EL and Meier P (1958) A non-parametric estimate from incomplete observations. *J Am Stat Assoc* **53**: 457–480

Kjonniksen I, Rye PD and Fodstad Ø (1994) Helix pomatia agglutinin in human tumour cell lines: correlation with pulmonary metastases in nude mice. *Br J Cancer* **69**: 1021–1024

Leathem AJ and Brooks SA (1987) Predictive value of lectin binding on breast cancer recurrence and survival. *Lancet* **I**: 1054–1056

Leathem A, Dokal I and Atkins N (1985) Lectin binding to normal and malignant breast tissue. *Diagn Histopathol* **6**: 171–180

Marks R and Kopf AW (1995) Cancer of the skin in the next century. *Int J Dermatol* **34**: 445–447

Mitchell BS and Schumacher U (1999) The use of the lectin *Helix pomatia agglutinin* (HPA) as a prognostic indicator and as a tool in cancer research. *Histol Histopathol* **14**: 217–226

Orfanos CE, Jung EG, Rassner G, Wolff HH and Garbe C (1994) Position paper of the Melanoma Committee of the German Dermatological Society on cutaneous malignant melanoma, with recommendations for diagnosis, treatment and follow up. Status 1993/94. *Hautarzt* **45**: 285–291

Schumacher U, Higgs D, Loizidou M, Pickering R, Leathem A and Taylor I (1994) Helix Pomatia Agglutinin Binding Is a Useful Prognostic Indicator In Colorectal Carcinoma. *Cancer* **74**: 3104–3107

Schumacher U, Adam E, Brooks SA and Leathem AJ (1995) Lectin binding properties of human breast cancer cell lines and human milk with particular reference to *Helix pomatia* agglutinin. *J Histochem Cytochem* **43**: 275–281

Shirashi T, Atsumi S and Yatani R (1992) Comparative study of prostatic carcinoma bone metastasis among Japanese in Japan and Japanese Americans and Whites in Hawaii. *Adv Exp Med Biol* **324**: 7–16

Tao TW and Burger MM (1982) Lectin-resistant variants of mouse melanoma cells. I. Altered metastasizing capacity and tumorigenicity. *Int J Cancer* **29**: 425–430

Walker RA (1993) *Helix pomatia* and prognosis of breast cancer. *Br J Cancer* **68**: 453–454

Yoshida Y, Okamura T and Shiakusa T (1993) An immunohistochemical study of *Helix pomatia* agglutinin binding on carcinomas of the oesophagus. *Surg Gynecol Obstet* **177**: 299–302

Zhang L, Zhou W, Velculescu VE, Kern SE, Hruban RH, Hamilton SR, Vogelstein B and Kinzler KW (1997) Gene expression profiles in normal and cancer cells. *Science* **276**: 1268–1272

*© 2001 Cancer Research Campaign* 
